# Hedgehog Pathway Blockade Inhibits Melanoma Cell Growth *in Vitro* and *in Vivo*

**DOI:** 10.3390/ph6111429

**Published:** 2013-11-11

**Authors:** Kathryn E. O’Reilly, Eleazar Vega-Saenz de Miera, Miguel F. Segura, Erica Friedman, Laura Poliseno, Sung Won Han, Judy Zhong, Jiri Zavadil, Anna Pavlick, Eva Hernando, Iman Osman

**Affiliations:** 1 Department of Dermatology, New York University School of Medicine, New York, NY 10016, USA; E-Mails: koreilly6@gmail.com (K.E.O.); Eleazar.Vega-SaenzdeMiera@nyumc.org (E.V.-s.M.); 2 Department of Pathology, New York University School of Medicine, New York, NY 10016, USA; E-Mails: Miguel.segura@vhir.org (M.F.S.); ZavadilJ@iarc.fr (J.Z.); Eva.Hernando-Monge@nyumc.org (E.H.); 3 Laboratory of Translational Research in Childhood Cancer, Vall d’Hebron Research Institute, Barcelona 08035, Spain; 4 Department of Surgery, New York University School of Medicine, New York, NY 10016, USA; E-Mail: efriedman324@gmail.com; 5 Oncogenomics Unit, Core Research Laboratory, Istituto Toscano Tumori, Pisa 56124, Italy; E-Mail: Laura.poliseno@gmail.com; 6 Department of Population Health, New York University School of Medicine, New York, NY 10016, USA; E-Mails: sungwonhan2@gmail.com (S.W.H.); Judy.Zhong@nyumc.org (J.Z.); 7 NYU Center for Health Informatics and Bioinformatics, New York University Langone Medical Center, New York, NY 10016, USA; 8 International Agency for Research on Cancer, 69372 Lyon Cedex 08, France; 9 Department of Medicine, New York University School of Medicine, New York, NY 10016, USA; E-Mail: Anna.Pavlick@nyumc.org

**Keywords:** melanoma, hedgehog, Smoothened, GLI2, PTCH1

## Abstract

Previous reports have demonstrated a role for hedgehog signaling in melanoma progression, prompting us to explore the therapeutic benefit of targeting this pathway in melanoma. We profiled a panel of human melanoma cell lines and control melanocytes for altered expression of hedgehog pathway members and determined the consequences of both genetic and pharmacological inhibition of the hedgehog pathway activator Smoothened (SMO) in melanoma, both *in vitro* and *in vivo*. We also examined the relationship between altered expression of hedgehog pathway mediators and survival in a well-characterized cohort of metastatic melanoma patients with prospectively collected follow up information. Studies revealed that over 40% of the melanoma cell lines examined harbored significantly elevated levels of the hedgehog pathway mediators *SMO*, *GLI2*, and *PTCH1* compared to melanocytes (*p <* 0.05). SMO inhibition using siRNA and the small molecule inhibitor, NVP-LDE-225, suppressed melanoma growth *in vitro*, particularly in those cell lines with moderate SMO and GLI2 expression. NVP-LDE-225 also induced apoptosis *in vitro* and inhibited melanoma growth in a xenograft model. Gene expression data also revealed evidence of compensatory up-regulation of two other developmental pathways, Notch and WNT, in response to hedgehog pathway inhibition. Pharmacological and genetic SMO inhibition also downregulated genes involved in human embryonic stem cell pluripotency. Finally, increased SMO expression and decreased expression of the hedgehog pathway repressor *GLI3* correlated with shorter post recurrence survival in metastatic melanoma patients. Our data demonstrate that hedgehog pathway inhibition might be a promising targeted therapy in appropriately selected metastatic melanoma patients.

## 1. Introduction

Melanoma incidence is quickly rising, with an estimated lifetime risk of 1 in 50 [1,2,3]. The average survival for a patient with metastatic disease is currently only 7 months [3,4]. Targeted therapies, such as B-RAF mutant kinase inhibitors, have shown dramatic initial responses in clinical trials, but resistance to single agent therapy inevitably ensues [5,6]. The CTLA-4 inhibitor, ipilimumab, induces more durable responses, but in just 20% of metastatic melanoma patients, and biomarkers for predicting response to this targeted agent are currently lacking [7,8]. Thus, despite recent advances, melanoma remains a clinical dilemma of considerable magnitude. We hypothesized that aberrantly activated developmental pathways may play an important role in melanoma. In this regard, several groups, including ours, have implicated the Notch pathway in melanoma progression [9,10]. Notch inhibitors currently in clinical trials have, however, shown limited efficacy in melanoma and gastrointestinal side effects remain an obstacle in it is clinical development [11,12,13].

Over the past five years, three independent groups have described the importance of yet another embryonic signaling cascade, the hedgehog pathway, in melanoma progression [14,15,16]. The hedgehog pathway is crucial in the embryo, ensuring proper spatiotemporal development. Physiologic hedgehog pathway activation occurs when secreted hedgehog ligand (HH) binds to the membrane associated Patched 1 (PTCH1) receptor, leading to activation of the Smoothened G-protein coupled receptor-like receptor (SMO) [17]. SMO signaling activates the GLI family zinc finger (GLI) transcription factors and results in transcription of target genes which include mediators of the pathway such as GLI1, PTCH1, cyclins D&E, and insulin-like growth factor binding proteins. While GLI1 and GLI2 mediate transcription of hedgehog target genes, GLI3 is a transcriptional repressor, leading to downregulation of hedgehog target genes. Aberrant activation of the pathway in adult tissue has been implicated in the pathogenesis of several cancers, and hedgehog signaling has been reported as a cancer stem cell pathway, mediating epithelial-to-mesenchymal transition via downregulation of E-cadherin [18,19,20,21,22,23,24]. Although no mutations in the hedgehog pathway have been described in melanoma, previous studies have demonstrated that hedgehog signaling is required for melanoma growth and metastasis in transgenic and xenograft mouse models [14,15]. Two studies also described high levels of GLI1 and GLI2 transcription factor mRNA in human metastatic melanoma tissue as compared to primary melanoma tissue [15,16]. Hedgehog pathway activity has also been shown to be necessary for the self-renewal of melanoma-initiating cells *in vitro* [25]. Most recently, the SMO antagonist NVP-LDE-225, (Novartis Pharma, AG, Basel, Switzerland) has been shown to inhibit melanoma growth both *in vitro* and *in vivo* [26]. These studies do not, however, examine the association between hedgehog pathway activity in melanoma and patient survival. In our study, we show that NVP-LDE-225, an oral hedgehog pathway inhibitor currently in a Phase II clinical trial, inhibits melanoma cell growth *in vitro* and *in vivo*, and we report, for the first time, the association of aberrant hedgehog pathway activity with decreased survival in melanoma patients.

## 2. Experimental

### 2.1. Human Melanoma Cell Lines

Primary melanoma lines WM35, WM1552c, WM1575, WM98.1, WM115, WM278, WM-793, WM-853, WM-902, WM-983, WM-1366, WM1361A, and WM3248 were purchased from the Wistar Institute (Philadelphia, PA, USA) and cultured in Mel 2% medium [27]. SkMel29, SkMel85, SkMel94, SkMel100, SkMel103, SkMel147, SkMel173, SkMel187, SkMel192, SkMel197 were kindly provided by Dr. Alan Houghton (MSKCC), and A375, Lu451, and 501 Mel were purchased from ATCC and cultured in DMEM supplemented with 10%FBS and 1% penicillin/streptomycin.

### 2.2. Data Mining of mRNA Microarray of Melanoma Cell Lines and qRT-PCR Confirmation

The U133A 2.0 Affymetrix array was performed on four melanocyte controls (two normal human melanocytes and two immortalized melanocytes), four primary and 14 metastatic cell lines as described in reference [28]. A curated gene signature of hedgehog pathway activation containing 77 distinct genes involved in hedgehog signaling was generated with the Broad Institute Molecular Signature Database and gene expression of these markers was analyzed in each of the 22 melanoma lines (including four primary and 14 metastatic melanoma lines) and melanocytes using Genespring GX software (Agilent, Santa Clara, CA, USA). Hierarchical clustering generated with Genespring GX software was used to create a heat map of hedgehog pathway gene expression. Increased levels of hedgehog pathway members were confirmed via quantitative real time PCR (qRT-PCR). RT-PCR was performed using qScript cDNA Supermix (Quanta Biosciences, Gaithersburg, MD, USA) and qPCR performed with Absolute Blue qPCR Sybr Green Mix (Thermo Fisher Scientific, Waltham, MA, USA). qPCR data analysis was performed using the ABI 7900HT (Applied Biosystems, Carlsbad, CA, USA) sequence detection instrument and threshold cycle values converted to relative transcript levels using the comparative Ct Method.

### 2.3. Data Mining of mRNA Microarray of Melanoma Cell Lines and qRT-PCR Confirmation, mRNA Microarray of Metastatic Melanoma Specimens

Using the previously published Human Genome U133A 2.0 Affymetrix expression profile of 44 metastatic melanoma samples [29] from patients followed clinically for a median of 35.4 months as well as the Human Genome U133 Plus 2.0 expression profile of 30 additional metastatic melanoma samples that we recently generated, we examined whether the canonical hedgehog pathway mediators *SHH*, *GLI1*, *GLI2*, *GLI3*, *SMO*, or *PTCH1* were associated with significantly decreased post-recurrence survival. RNA was isolated from the 30 additional metastatic melanoma specimens using the RNAeasy Kit (Qiagen Sciences, Germantown, MD, USA).The 30 additional metastatic melanoma patients were identified through the Interdisciplinary Melanoma Cooperative Group database at New York University School of Medicine (19 male, 11 female; median age, 63.4 years). Of the 30 specimens from 30 patients, 11 were lymph node metastases, 15 were skin metastases, and four were visceral metastases. The median follow-up time for the cohort from the time of primary diagnosis to last follow-up date was 70.08 months. The study was approved by the New York University Institutional Review Board, and all patients signed informed consent before enrollment. Relevant clinicopathologic, demographic, and survival data were recorded for all patients.

### 2.4. Statistical Analysis

Descriptive statistics were calculated for baseline demographic and clinicopathologic characteristics. Cox proportional hazards model dichotomized at the median expression value was used to examine the association between hedgehog pathway mediatory transcript levels and post-recurrence survival (time from first recurrence to death). Kaplan-Meier curves were generated using Graph Pad Prism 5.0 Software (LaJolla, CA, USA).

### 2.5. *In Vitro* Cell Proliferation Assays of Cyclopamine, NVP-LDE-225, and Vemurafenib

The indicated cell lines were seeded at a density of 1 × 10^4^ cells per well in a 12-well dish in triplicate in DMEM medium*.* The day after (day 0), the medium was replaced, and DMSO, cyclopamine (Toronto Research Chemicals, North York, ON, Canada), the oral Smoothened inhibitor in Phase II clinical trials, NVP-LDE-225, (Novartis Pharma AG), or Vemurafenib (ChemieTek, Indianapolis, IN, USA) at indicated concentrations were added. At the indicated time points (3–12 days), cells were fixed in 10% formalin solution and stored in PBS at 4 °C. After the final time point, all the plates were stained with crystal violet. After color elution with 10% acetic acid, optical density was read at 590 nm. A representative curve of three independent experiments is reported.

### 2.6. Cell Cycle and Apoptosis Analysis

5 × 10^5^ A375 cells were plated in 10 cm plates, and 24 h later were treated with DMSO or 5 µM NVP-LDE-225. After 72 h of treatment, cells were trypsinized, washed with PBS, and fixed in 70% ethanol. Prior to FACS analysis, fixed cells were stained with propidium iodide in PBS (25 µg/mL) containing 250µg/ml RNase A. Pan-caspase activation and changes in mitochondrial potential were determined in A375 and WM3248 cells after 72 h of treatment with 10 µM NVP-LDE-225 using the dual sensor MitoCasp™ assay (Cell Technology, Mountain View, CA, USA) according to the manufacturer’s protocol. Stained cells were evaluated in an LSRII flow cytometer and analyzed using Flowing Software version 2.4 (Perttu Terho, Turku Centre for Biotechnology, Turku, Finland).

### 2.7. siRNA Assays

2–4 × 10^4^ melanoma cells were plated in antibiotic free medium into each well of a 12 well plate. 18 h later, 40 pmol of ON-Target plus SMPARTpool Human SMO (L-005726-00-0005), Human GLI1 (L-003896-00-0005), or Human GLI2 (L-006468-00-0005) siRNA (Thermo Scientific Dharmacon, Lafayette, CO, USA) diluted in Opti-Mem I reduced serum media were transfected into the cells using Lipofectamine 2000 (Invitrogen, Grand Island, NY, USA). The medium was changed after 6 h and cells were trypsinized for quantitative real time PCR or stained with crystal violet at indicated time points.

### 2.8. Xenograft Assay of A375 Cells

1.5 × 10^6^ A375 cells were injected into the flank of NOD/Scid/IL2 gamma receptor -/- (NOG) mice (n = 20). Once tumors were palpable (6 days after injection), mice were randomized into two groups and vehicle (n = 10) or NVP-LDE-225 was administered orally at 60 mg/kg/day. After one week of treatment, tumors were excised, weighed, and RNA was isolated from tumors for RT-PCR. Effective pathway inhibition was confirmed with qPCR of PTCH1 transcript levels in the tumors of NVP-LDE-225 treated mice as compared to vehicle treated mice.

### 2.9. Pathway Analysis of SMO Inhibition Transcriptional Profile

The transcriptional profile of NVP-LDE-225 and SMO siRNA treated WM3248 and A375 melanoma cells was generated using a Human Genome U133 Plus 2.0 genechip. The WM3248 and A375 cell lines were treated for 24 h with either 5 µM NVP-LDE-225 or SMO siRNA. The significantly downregulated canonical signaling pathways in SMO inhibitor treated WM3248 and A375 cell lines, as compared to DMSO or non-targeting siRNA treated cells, respectively, were generated with Ingenuity Pathway Analysis software (Ingenuity Systems, Inc., Redwood City, CA). Canonical pathways analysis identified the pathways from the IPA library of canonical pathways that were most significant to the data set. Genes from the data set that met the fold change cutoff of 1.2 and were associated with a canonical pathway in the Ingenuity Knowledge Base were considered for the analysis. The significance of the association between the data set and the canonical pathway was measured in 2 ways: (1) A ratio of the number of molecules from the data set that map to the pathway divided by the total number of molecules that map to the canonical pathway is displayed. (2) Fisher’s exact test was used to calculate a p‐value determining the probability that the association between the genes in the dataset and the canonical pathway is explained by chance alone.

## 3. Results and Discussion

### 3.1. Expression of Hedgehog Pathway Members is Elevated in Melanoma Cell Lines compared to Melanocytes

**Figure 1:**
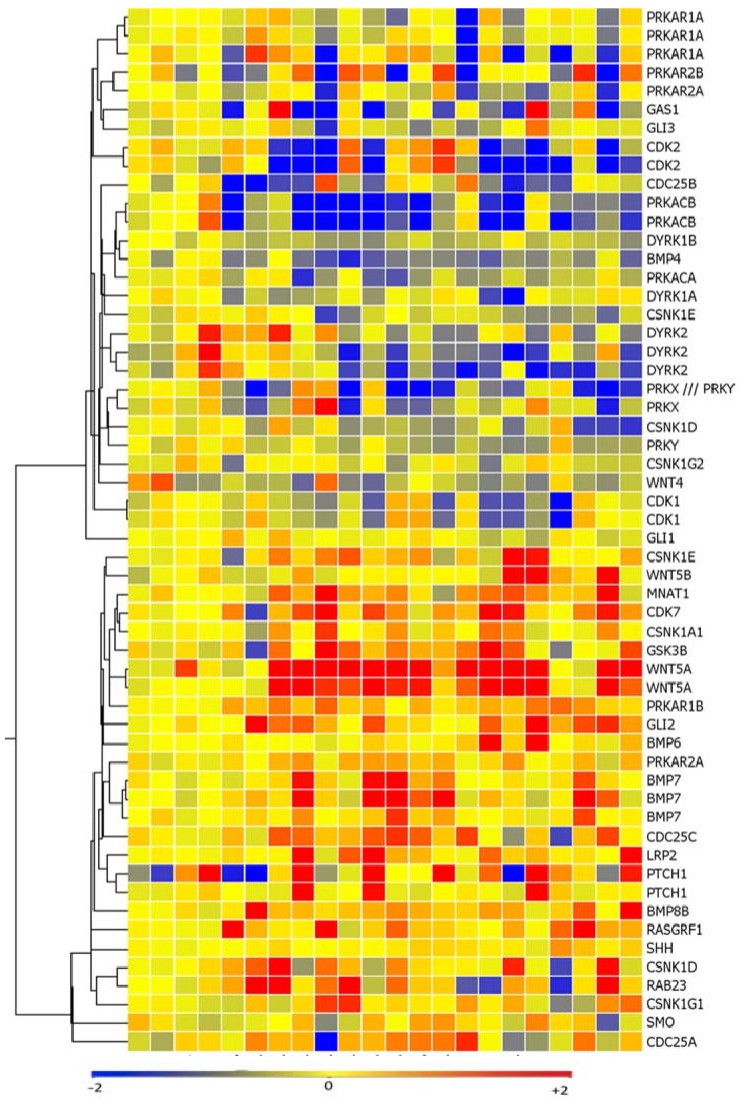
Hedgehog pathway members are upregulated in a subset of melanoma cell lines as measured via affymetrix microarray analysis. SMO, GLI2, PTCH1, and WNT5a are upregulated, while negative regulators of hedgehog signaling such as protein kinase A (PRKA) and dual specificity tyrosine-phosphorylation-regulated kinase 2 (DYRK2) are downregulated as compared to melanocytes.

We analyzed the expression of 77 genes from a curated gene signature of hedgehog pathway activation using our microarray expression profile of 18 melanoma cell lines and four melanocyte controls [28]. Array expression values of *WNT5A* and *GLI2* were found to be significantly higher than melanocytes in over 50% of the melanoma cell lines, (14/18 and 14/18 cell lines, respectively) (Figure 1) (*p* < 0.05).

**Figure 2:**
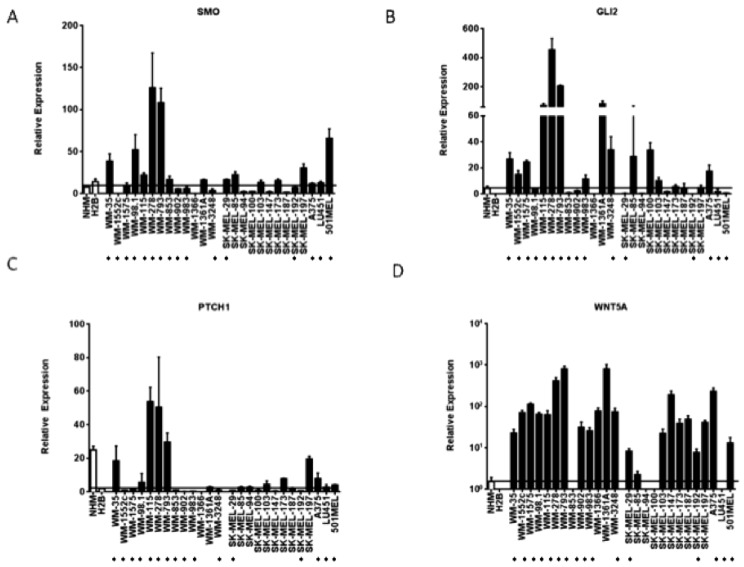
Hedgehog pathway members are upregulated in a subset of melanoma cell lines. (**A**–**D**) Elevated transcript levels of SMO, GLI2, PTCH1, and WNT5a detected by Affymetrix microarray analysis were confirmed by quantitative RT-PCR. Values represent the mean ± standard deviation of triplicate qPCRs from three different experiments. Open bars represent melanocytes controls, while the closed bars represent melanoma cell lines. The cell lines beginning with “WM” are primary melanoma cell lines, while the cells lines beginning with “SK-MEL” and the A375, LU451, and 501MEL cell lines are metastatic melanoma cell lines. (*) Indicates that the cell line is a V600E B-RAF mutant.

*SMO* and *PTCH1* expression levels were also higher in a subset of melanoma cell lines as compared to melanocytes (8/18 and 8/18 cell lines, respectively) (*p* < 0.05). There was also loss of expression of negative regulators of the hedgehog pathway such as protein kinase A (*PRKA*) and dual-specificity tyrosine-(Y)-phosphorylation regulated kinase 2 (*DYRK2*) in a subset of melanoma cell lines (13/18 and 12/18 cell lines, respectively) (*p* < 0.05). 11 cell lines expressed high levels of both WNT5a and GLI2 as compared to the melanocytes controls and 10/11 of these cell lines (91%) displayed loss of both DYRK2 and PRKA (Figure 1). The elevated expression of *PTCH1*, *SMO*, *GLI2*, and *WNT5A* detected with array technology was validated in an expanded melanoma cell line panel of 26 melanoma cell lines using quantitative real time PCR (Figure 2a–d).

**Figure 3:**
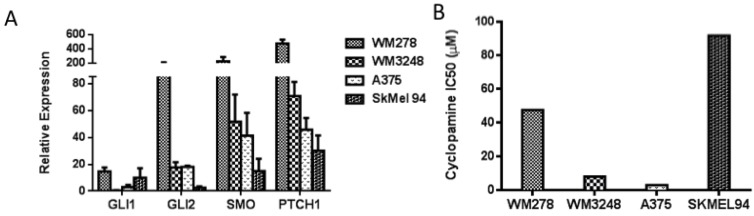
Melanoma cell lines harboring modest expression of GLI2 and SMO were more sensitive to cyclopamine as compared to those cell lines with overexpression or absence of GLI2 and SMO. (**A**) Baseline levels of GLI1, GLI2, SMO, and PTCH1 were measured by qRT-PCR in four melanoma cell lines. (**B**) The A375 and WM3248 cell lines were sensitive to cyclopamine (IC_50_ < 10 μM), whereas the WM278 and SkMel94 cell lines were resistant to cyclopamine (IC_50_ > 45 μM). In A, values represent the mean ± standard deviation from a representative experiment performed three times with similar results. In B the values represent the mean from a representative experiment performed three times with similar results.

As compared to normal or immortalized melanocytes, *GLI2* mRNA was found to be elevated in 13/26 (50%) melanoma cell lines and *SMO* expression was elevated in 13/26 (50%) cell lines. Six of the cell lines overexpressing GLI2 as compared to melanocytes concomitantly overexpressed SMO. The GLI2 transcriptional target *WNT5A* was found to upregulated in 22/26 (85%) melanoma cell lines as compared to melanocyte controls. Finally, the hedgehog pathway transcriptional target *PTCH1* was elevated in 10/26 (39%) melanoma cell lines as compared to immortalized melanocytes. Of note, in our microarray analyses, *GLI1* was not found upregulated in our panel of melanoma cells as compared to melanocytes. *GLI1* was found to be expressed at much lower levels than *GLI2*, *SMO*, or *PTCH1* mRNA as measured by quantitative qRT-PCR (Figure 3a). The increased expression of two effectors of hedgehog signaling, such as *GLI2* and *SMO*, as well as transcriptional targets of hedgehog signaling like *PTCH1* and *WNT5A*, suggests that half of the melanoma cell lines tested harbor upregulated hedgehog pathway activity as compared to control melanocytes.

### 3.2. SMO Inhibition Decreases Melanoma Cell Proliferation *in Vitro*

We then examined the effect of hedgehog pathway inhibition on melanoma growth. The small molecule SMO inhibitor cyclopamine decreased cell proliferation after 72 h of treatment in the A375 and WM3248 cell lines, which demonstrated modestly elevated *GLI2*, *SMO*, *PTCH1* expression (between 10 and 60 fold increase as measured by qPCR) (Figure 3b). Cyclopamine demonstrated weaker antiproliferative effects in the SKMel94 cell line with low *GLI2*, *SMO*, *PTCH1* expression (<5 fold increase), and in the WM278 cell line, which harbors a one copy GLI2 and two copy SMO allele gain as detected by a DNA SNP array [26] and overexpresses GLI2 and SMO relative to melanocytes (fold increase for both GLI2 and SMO > 100) and the majority of other melanoma cell lines in our panel (IC_50_ values of 47.5 and 91.9 μM, respectively) (Figure 3b). Interestingly, we observed that cell lines with modest expression of pathway members were more sensitive to SMO inhibition than those with either very low (less than 20 fold increase) or very high expression (over 150 fold increase) of GLI2, SMO, and PTCH1.

**Figure 4:**
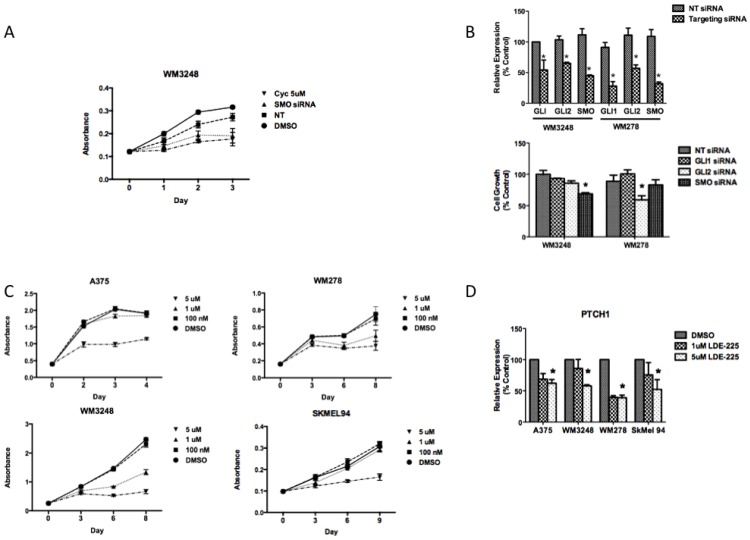
Smoothened inhibition has antiproliferative effects in melanoma *in vitro*. (**A**) siRNA against *SMO* demonstrates a similar antiproliferative effect as 5 µM cyclopamine after three days in the WM3248 cell line, as measured by crystal violet staining. (**B**) Knockdown of hedgehog pathway mediators using siRNA to target *GLI1*, *GLI2*, or *SMO* was performed in the WM3248 and WM278 cell lines. In the WM3248 cell line, which was sensitive to cyclopamine, siRNA to SMO has better antiproliferative effects than GLI1 or GLI2 inhibition. In the WM278 cell line, which is resistant to cyclopamine, and harbors amplification of SMO and GLI2, GLI2 inhibition decreases proliferation more than inhibition of either GLI1 or SMO (*, *p* < 0.05). (**C**) The SMO inhibitor NVP-LDE-225 inhibits melanoma cell growth in the A375, WM278, WM3248, and SkMel94 cell lines. (**D**) 5 µM NVP-LDE-225 inhibits hedgehog signaling *in vitro* as evidenced by decreased PTCH1 transcript levels after 24 h of treatment (*, *p* < 0.05). In A–C, values represent the mean ± standard deviation from a representative experiment performed three times with similar results. In D, the values represent the mean ± standard deviation of triplicate qPCRs from three different experiments.

To account for potential off-target effects of small molecule SMO inhibition, we employed SMO siRNA to determine whether genetic interference with the hedgehog pathway would also inhibit growth. Transient transfection of siRNA against SMO demonstrated a similar antiproliferative effect at 72 h as three days of 5μM cyclopamine treatment in the WM3248 cell line (Figure 4a). siRNA to SMO inhibited proliferation in the WM3248 cell lines better than GLI1 or GLI2 silencing (Figure 4b). In the WM278 cell line, neither SMO nor GLI1 silencing inhibited proliferation as compared to non-targeting siRNA transfected cells (Figure 4b).

In WM278, GLI2 silencing inhibited proliferation better than inhibition of either SMO or GLI1. The weak antiproliferative effect of SMO silencing in the WM278 cell line suggests that overexpression of GLI2, a downstream hedgehog pathway effector, may render melanoma cells resistant to SMO inhibition. SMO silencing by siRNA mimicked the effect of cyclopamine in melanoma cells, with antiproliferative effects in cells that show an IC_50_ of less than 10 μM and a lack of antiproliferative effect in cells that displayed an IC_50_ greater than 45 μM (Figure 4b).

### 3.3. NVP-LDE-225 Inhibits Melanoma Cell Proliferation and Induces Apoptosis *in Vitro*

As cyclopamine’s unfavorable pharmacokinetic properties preclude its use in humans, we investigated the effects of the clinically relevant SMO inhibitor, NVP-LDE-225, on melanoma cell proliferation. The small molecule NVP-LDE-225 is a well-tolerated oral SMO inhibitor currently in Phase II clinical trials in advanced basal cell carcinoma. We found that NVP-LDE-225 decreased melanoma cell proliferation both *in vitro* and *in vivo*. NVP-LDE-225 inhibited the proliferation of A375, WM3248, WM278, and SkMel 94 cell lines after 3-6 days of treatment (Figure 4c). NVP-LDE-225 effectively inhibited hedgehog pathway signaling, as indicated by a decrease in PTCH1 expression after 24 h of treatment in all four cell lines tested (Figure 4d).

**Figure 5:**
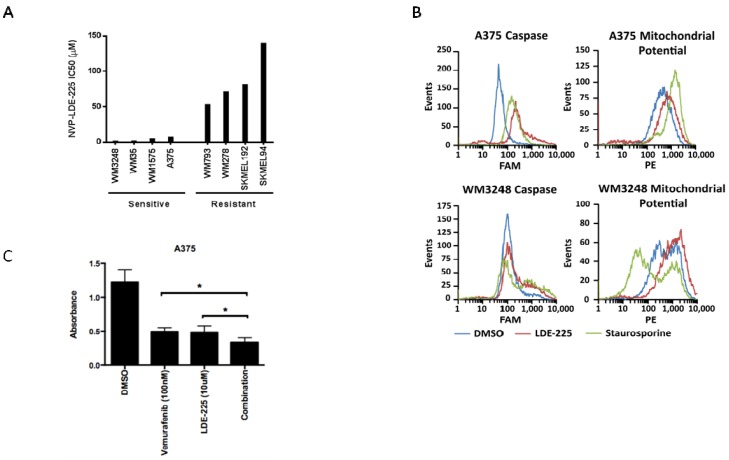
The SMO inhibitor NVP-LDE-225 inhibits proliferation in primary and metastatic melanoma cell lines *in vitro*. (**A**) Primary and metastatic melanoma cell lines demonstrate variable sensitivity to NVP-LDE-225. Cell lines harboring modest expression of GLI2 and SMO are more sensitive to NVP-LDE-225 (IC_50_ < 15 µM) as compared to those cell lines with overexpression of GLI2 and SMO (WM278 and WM793) or lack of pathway activity (SkMel94 and SkMel100) (IC_50_ > 50 µM). (**B**) 10 µM NVP-LDE-225 induces apoptosis in A375 and WM3248 cells as detected by an increase in pan-caspase positive cells and in cells with loss of mitochondrial membrane potential after 72 h of treatment. (**C**) Combination LDE-225 (10 µM) and vemurafenib (100 nM) inhibits proliferation better than either single agent or vehicle after 72 h of treatment (*, *p* < 0.003). In A–B, values represent the mean from a representative experiment performed three times with similar results. In C, values represent the mean ± standard deviation from a representative experiment performed three times with similar results.

As observed with cyclopamine treatment, cell lines with moderate expression of hedgehog pathway mediators GLI2 and SMO were more sensitive to NVP-LDE-225 than those cell lines harboring amplification and/or overexpression of hedgehog family members (Figure 5a). IC_50_s for WM3248, WM35, WM1575, and A375 cell lines with moderate expression of both GLI2 and SMO ranged from 1.4–6.8 μM, while IC_50_s for WM793 and WM278 which overexpress GLI2 and SMO were over 50 μM. The SkMel 192 and SkMel 94 cell lines which lack expression of SMO and GLI2 were most resistant to NVP-LDE-225 with IC_50_s of 81.38 and 139.3 μM, respectively (Figure 5a).

In order to ascertain how the inhibition of the hedegehog pathway impacts on cell number, we analyzed the cell cycle profile of melanoma cells sensitive to NVP-LDE-225. Cell cycle profiles revealed no major differences between NVP-LDE-225 treated A375 and WM3248 cells *versus* control (data not shown). However, cell death analysis using a pan-caspase detection assay and a mitochondrial potential assay revealed an increased percentage of apoptotic cells after 72 h of NVP-LDE-225 treatment in both the A375 and WM3248 cells (Figure 5b). As the four melanoma cell lines tested that were most sensitive to NVP-LDE-225 were all V600E B-RAF mutant cell lines (as confirmed by DNA sequencing in our laboratory), we examined a potential synergism between NVP-LDE-225 and the BRAF inhibitor vemurafenib. For that, A375 cells were treated with DMSO, NVP-LDE-225 (10 μM), the B-RAF inhibitor vemurafenib (100 nM), or the combination of both for three days. We found a 31% reduction in cell growth in the combination treated cells as compared to single agent treated cells (*p* = 0.0025) (Figure 5c).

**Figure 6:**
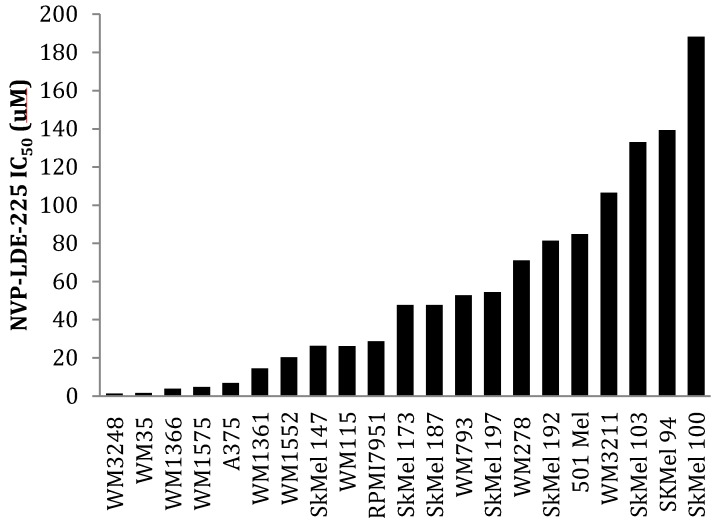
A panel of 18 melanoma cell lines demonstrates differential sensitivity to NVP-LDE-225. WM35, WM3248, WM1575, A375, WM1361, and WM1366 were sensitive to NVP-LDE-225 (IC_50_ < 15 µM). WM1552, SkMel147, WM115, and RPMI7951 demonstrated intermediate sensitivity to NVP-LDE-225 (IC_50_ 20 µM–30 µM), while the remaining 11 cell lines were resistant to the inhibitor (IC_50_ > 50 µM). The values represent the mean from a representative experiment performed three times with similar results.

We then examined the impact of BRAF mutation status on the response (or lack thereof) to hedgehog pathway inhibition. Our data revealed that 36% (6/16) of the V600E B-Raf mutant melanoma cell lines had modest expression of GLI2 and SMO as compared to melanocytes (Figure 2a,b). In our panel of V600E B-Raf mutant cell lines treated with NVP-LDE-225, we found that 36% (4/11: WM35, WM3248, WM1575, and A375) were sensitive to the inhibitor (IC_50_ < 10 µM) (Figure 6). 50% (2/4) of the N-Ras mutant cell lines (WM1366 and WM1361A) were sensitive to the NVP-LDE-225, while none of the three B-Raf wild type cell lines (SkMel 173, 187, and 197) were sensitive. Likewise, the c-kit mutant cell line WM3211 and the two V599K B-Raf mutant cell lines SkMel 94 and SkMel 100 were resistant to the inhibitor (IC_50_ > 50 µM, Figure 6). The four BRAF mutant cell lines sensitive to NVP-LDE-225 demonstrated moderate GLI2 and SMO expression, as did the sensitive N-Ras mutant cell line, WM1361A.

### 3.4. NVP-LDE-225 Inhibits Melanoma Growth *in Vivo*

**Figure 7:**
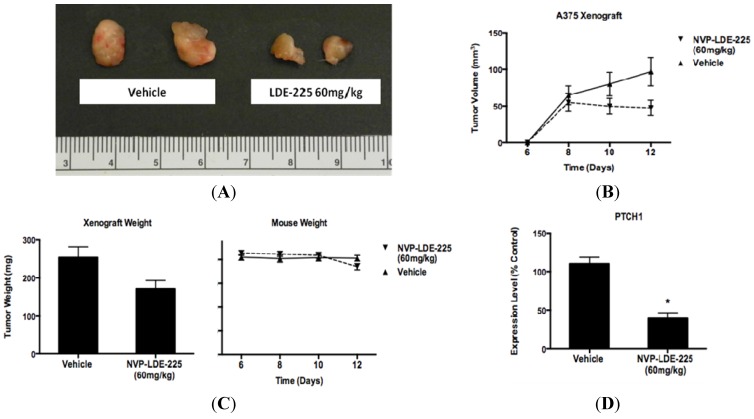
NVP- LDE-225 60 mg/kg inhibits melanoma cell proliferation *in vivo*. (**A**) One week of daily NVP-LDE-225 60 mg/kg administered by oral gavage to an A375 xenograft model in NOG mice inhibits melanoma growth better than vehicle. (**B**–**C**) Tumors in NVP-LDE-225 treated mice (n = 6) are of lower volume (*p* = 0.103) and weight (*p* = 0.0979) than the tumors in vehicle treated mice (n = 7). The mice in the vehicle and LDE-225 treated groups are of similar weight throughout the duration of treatment (*p* = 0.691). (**D**) NVP-LDE-225 inhibits the hedgehog pathway as measured by decreased PTCH1 transcript in the tumors of NVP-LDE-225 treated mice as compared to the transcript levels in the tumors of control treated mice, as measured by qRT-PCR (*, *p <* 0.003). In B–C, values represent the mean ± standard deviation of at least 6 animals per treatment group. In D, the values represent the mean ± standard deviation of triplicate qPCRs from the tumors of three mice from each treatment group.

As NVP-LDE-225 inhibited cell viability *in vitro* in a subset of our melanoma cell line panel, we wanted to determine whether oral administration of NVP-LDE-225 would inhibit melanoma growth *in vivo*. One week of daily 60mg/kg NVP-LDE-225 suspension delivered by oral gavage to NOG mice bearing A375 xenografts resulted in decreased tumor volume (*p* = 0.103) and tumor weight (*p* = 0.0979) as compared to the vehicle treated mice (Figure 7a–c). The average mouse weight in the treatment group was lower than in the vehicle group; however the difference was not statistically significant (Figure 7c). The treatment duration was limited to one week as four of the ten mice in the treatment group exhibited direct local GI toxicity secondary to the suspension formulation used for oral gavage, an adverse event not observed in human phase I studies of NVP-LDE-225 capsules. RNA was extracted from vehicle and NVP-LDE-225 tumors and PTCH1 expression was found to be 60% lower in the NVP-LDE-225 treated tumor as compared to vehicle-treated tumors (Figure 7d), supporting effective targeting of the HH pathway.

### 3.5. Increased SMO Levels are Associated with Decreased Survival, while Elevated GLI3 Levels are Associated with Better Survival in Metastatic Melanoma Patients

**Figure 8:**
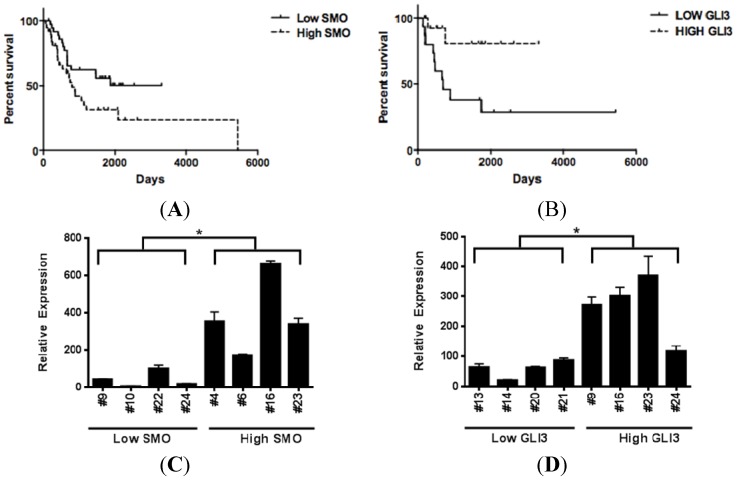
Hedgehog pathway activity is associated with decreased post-recurrence survival in metastatic melanoma patients. (**A**) Elevated SMO expression is associated with significantly shorter survival in metastatic melanoma patients (Hazard Ratio = 1.927, *p* = 0.0374). (**B**) Elevated GLI3 levels, a hedgehog pathway transcriptional repressor, is associated with increased survival in metastatic melanoma patients (Hazard Ratio = 0.15, *p* = 0.015). (**C**–**D**) Transcript levels of SMO and GLI3 in metastatic melanoma specimens are verified using qRT- PCR. Patient specimen numbers are listed on the x axis. Low and high expression levels of SMO and GLI3 were defined as below or above the median expression value, (*, *p <* 0.05).

As prior reports describe increased expression of hedgehog pathway members in metastatic melanoma specimens as compared to primary melanoma tissue, we sought to examine if there was an association between hedgehog pathway activity and survival. Microarray gene expression profiles from 44 metastatic melanoma specimens were examined for expression levels of *SHH*, *GLI1*, *GLI2*, *SMO*, *PTCH1*, *and WNT5* [29]. We then examined the association of these transcript levels with metastatic melanoma patient outcomes. In this cohort, elevated levels of SMO expression correlated with significantly decreased post-recurrence survival as compared to those patients expressing levels of SMO below the median expression value (HR = 2.85, *p* = 0.006). We isolated RNA from 30 additional metastatic melanoma specimens and performed U133 2.0 Plus Affymetrix array on these samples. Upon examining the pooled expression values from all 74 samples, there remained a statistically significant association between elevated SMO and decreased survival in the expanded cohort (Figure 8a, HR = 1.927, *p* = 0.0374). *SHH*, *GLI1*, *GLI2*, *PTCH1*, *and WNT5* were not associated with decreased survival in this expanded cohort. While the hedgehog pathway transcriptional repressor *GLI3* was not detected by the U133A platform in any of our first 44 melanoma specimens, the U133 2.0 Plus array analysis did detect *GLI3* transcript expression and revealed differential expression among the 30 metastatic melanoma specimens analyzed. Elevated expression of the hedgehog pathway transcriptional repressor *GLI3* (as defined as above the median expression level) in the 30 metastatic tumor specimens associated with better survival in metastatic melanoma patients (Figure 8b, HR = 0.15, *p* = 0.015). Microarray expression levels of *GLI3* and *SMO* in patient tumors were confirmed by quantitative real time PCR (Figure 8c,d).

### 3.6. SMO Inhibition Induces a Programmed Cell Death Gene Expression Signature and Downregulates Embryonic Stem Cell Pluripotency

To determine the mechanism of SMO inhibitor-induced apoptosis, we performed gene expression profiling of melanoma cells treated with NVP-LDE-225 and SMO siRNA treated melanoma cells. Microarray expression profiles of WM3248 and A375 cell lines treated with siRNA to SMO or the small molecule NVP-LDE-225 were compared to non-targeting siRNA or DMSO treated controls, respectively. In the WM3248 cell line, both genetic interference and NVP-LDE-225 treatment for 24 h led to downregulation of cell adhesion molecules claudin20 and ICAM2, as well as the metalloproteases BMP1 and MMP28, while mediators of programmed cell death such as caspase I, forkhead BoxO3 pseudogene, FOS-like antigen 2, and BCL2 interacting protein 3 were upregulated (*p <* 0.05). In the A375 cell line, both siRNA to SMO and NVP-LDE-225 downregulated the growth factor PDGF-alpha and the protein translation mediator eIF4a2, while upregulating the apoptosis mediator annexin A9 and the melanoma antigen MAGE-E1 (*p <* 0.05). Interestingly, in both the A375 and WM3248 cell lines, NVP-LDE-225 downregulated the Ras signaling adaptor GRAP (−1.22 averaged fold change, *p <* 0.05) and upregulated XAF1 (+1.2 averaged fold change, *p <* 0.05), a mediator of programmed cell death. In both cell lines tested, the notch ligand DLL3 (+1.23 averaged fold change, *p* = 0.041) and the WNT5a receptor RORA were upregulated by NVP-LDE-225 treatment (+1.34 averaged fold change, *p* = 0.0004).

**Figure 9:**
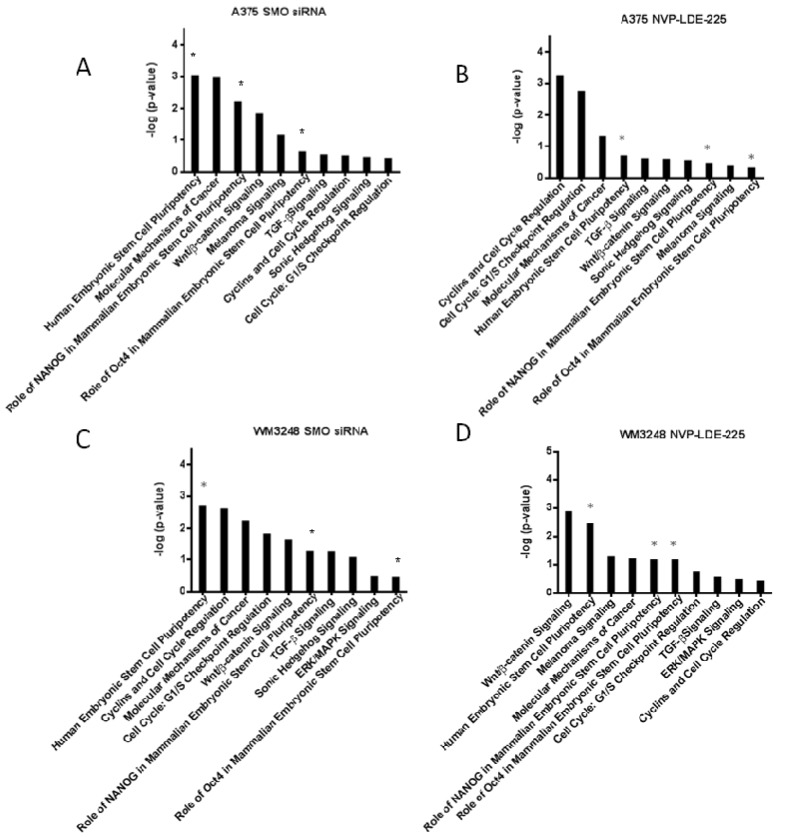
Ingenuity Canonical Pathway analysis reveals downregulation of stem cell pluripotency signaling and cell cycle regulators. (**A**–**D**) Downregulated IPA (Ingenuity Pathway Analysis) canonical pathways are shown for A375 and WM3248 cell lines treated with either 5 µM NVP-LDE-225 or SMO siRNA. The transcriptional profiles of the DMSO treated and non-targeting siRNA treated A375 and WM3248 cells were used as controls in the IPA analysis. The stem cell pathways that were downregulated by both SMO siRNA and NVP-LDE-225 in both cell lines are marked with an asterisk (*).

Ingenuity Pathway Analysis (IPA) was also performed on the transcriptional profile of NVP-LDE-225 and SMO siRNA treated melanoma cells. The pathways from the IPA canonical pathway library that were significantly downregulated by both SMO siRNA and NVP-LDE-225 in both the A375 and WM3248 cell lines are pictured in Figure 9. The IPA revealed downregulation of the Human Embryonic Stem Cell Pluripotency pathway in both SMO siRNA and NVP-LDE-225 treated WM3248 and A375 cells (*p <* 0.05). The role of NANOG in Mammalian Embryonic Stem Cell Pluripotency pathway and the role of Oct4 in Mammalian Embryonic Stem Cell Pluripotency pathway were also significantly downregulated in both SMO siRNA and inhibitor treated A375 and WM3248 cells (*p <* 0.05).

**Figure 10:**
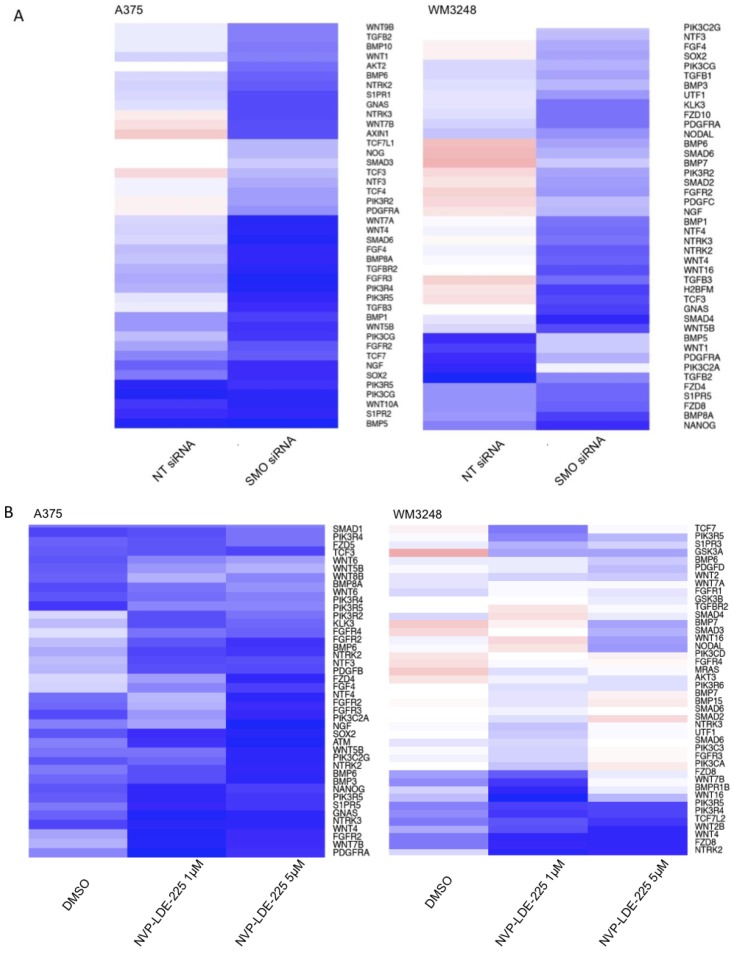
Genetic and Pharmacologic Inhibition of SMO downregulates mediators of stem cell pluripotency. (**A**) SMO siRNA downregulates expression of IPA human stem cell pluripotency pathway members as compared to non-targeting (NT) siRNA treated WM3248 and A375 cells. (**B**) NVP-LDE-225 downregulates expression of IPA human stem cell pluripotency pathway members as compared to DMSO- treated WM3248 and A375 cells.

A heatmap of differentially expressed genes from the IPA human stem cell pluripotency pathway in SMO siRNA and NVP-LDE-225- treated cells as compared to their respective controls is pictured in Figure 10a,b. Our IPA analysis also showed that various apoptosis pathways were upregulated by SMO inhibition (Table 1). SMO siRNA upregulated the Death Receptor Signaling Pathway and MYC Mediated Apoptosis Signaling (*p <* 0.05). NVP-LDE-225 upregulated the Death Receptor Signaling Pathway, MYC Mediated Apoptosis Signaling, and Apoptosis Signaling in melanoma cell lines (*p <* 0.05).

**Table 1:** Genetic and Pharmacologic Inhibition of SMO upregulates apoptosis signaling. Both SMO siRNA and NVP-LDE-225 upregulate apoptosis pathways in WM3248 and A375 cell lines.

	A375	WM3248
SMO siRNA	-Death Receptor Signaling Pathway	-Myc Mediated Apoptosis Signaling
NVP-LDE-225	-Apoptosis Signaling	-Death Receptor Signaling Pathway
	-Myc Mediated Apoptosis Signaling	-Myc Mediated Apoptosis Signaling

## 4. Conclusions

Here we show, for the first time, that elevated levels of hedgehog pathway components correlate with worse survival in melanoma patients. We also demonstrate that NVP-LDE-225, a small molecule SMO inhibitor already in clinical trials in other solid tumors, inhibits the hedgehog pathway effectively and has antitumor activity against B-RAF mutant melanoma both *in vitro* and *in vivo*. Our findings that NVP-LDE-225 inhibits proliferation and induces apoptosis in melanoma cell lines agree with those of Jalili *et al.* However, in contrast to their study, we observed a more dramatic antiproliferative effect in B-RAF mutant cell lines as compared to B-RAF wild type cells and we did not observe a cell cycle arrest upon NVP-LDE-225 treatment. The relative resistance of B-RAF wild type melanoma cell lines and the lack of cell cycle arrest observed in our study may be secondary to the different melanoma cell lines used. While Jalili *et al.* observed cell cycle arrest in NVP-LDE-225 treated UACC 257 and LOX IMVI cell lines, we observed no cell cycle alterations in NVP-LDE-225 treated WM3248 and A375 melanoma cell lines. Similarly, while Jalili *et al.* demonstrated sensitivity of MEWO and MEL FH wild type B-RAF cell lines to NVP-LDE-225, the three wild type B-RAF cell lines (SkMel173, SkMel187, and SkMel197) used in our study were resistant to NVP-LDE-225, with IC_50_s > 50 µM. Finally, the more robust *in vivo* antitumor response observed in Jalili *et al.*’s xenograft model may be secondary to the direct intratumoral delivery of NVP-LDE-225 in their animal model as compared to the oral gavage delivery method used in our study.

Our observations suggest that hedgehog pathway inhibitors may be an additional therapeutic strategy against B-RAF mutant melanoma. As activated RAS/MAPK signaling has been shown to induce hedgehog pathway activity via translocation of GLI1 and GLI2 into the nucleus, it is possible that B-RAF mutation is augmenting hedgehog pathway activity in our melanoma cell lines and that inhibiting both B-RAF and the hedgehog pathway may result in an enhanced therapeutic effect. Our data demonstrate a modest but significant effect of combining B-RAF and hedgehog inhibitors, but additional studies of the dosage and sequence of administration *in vivo* are necessary to better define a clinically useful regimen. Whether SMO inhibition might be effective in vemurafenib resistant B-RAF mutant tumors is currently under investigation in our laboratory.

Although other groups have reported upregulation of hedgehog pathway members in metastatic tissue as compared to primary specimens, this is the first report of an association of SMO levels with significantly decreased survival in metastatic melanoma patients. The protective effect of elevated GLI3 levels underscores the role of hedgehog pathway activation in more aggressive disease. We also report that a clinically relevant inhibitor already in phase II clinical trials inhibits melanoma growth *in vitro* and *in vivo*. In ongoing clinical trials in advanced basal cell carcinoma, this daily dosed oral inhibitor has been well tolerated with little toxicity. Thus, our findings could be readily translated into clinical trials of a SMO inhibitor in combination with vemurafenib in patients whose B-RAF mutant tumors express GLI2 and SMO at moderate levels.

Ingenuity pathway analysis revealed that silencing of the hedgehog pathway by either chemical or genetic means significantly inhibited the expression of human embryonic stem cell pluripotency related genes. This result indicates that SMO inhibitors may effectively target the melanoma stem cell compartment and supports a recent study showing that melanoma-initiating cells require hedgehog signaling in order to self-renew [25]. In order to achieve a durable antitumor response, it may be necessary to not only target mutated oncogenic pathways in melanoma (*i.e.*, B-RAF), but also the embryonic signaling pathways promoting self renewal in a subset of melanoma cells. Inhibition of melanoma initiating cell self-renewal might extend the durability of the robust, albeit short-lived, antitumor responses achieved with vemurafenib in metastatic melanoma patients.

NVP-LDE-225 induced a programmed cell death gene expression profile in both cell lines tested, and also paradoxically upregulated the notch ligand *DLL3* and the WNT5 receptor *ROR* in both the A375 and WM3248 cell lines. As both of the cell lines tested demonstrated upregulation of the programmed cell death mediator *XAF1* in response to both small molecule and genetic SMO inhibition, this protein is likely a key mediator in hedgehog inhibitor-induced apoptosis. The upregulation of the notch ligand *DLL3* and *WNT5* that we observed in our microarray analysis indicate a compensatory upregulation of alternative stem cell signaling pathways in response to SMO inhibition. Compensatory upregulation of alternate stem cell pathways is a phenomenon previously reported with Notch inhibitors in neural tumors [30]. Schreck et al demonstrated that single agent Notch inhibition activated hedgehog signaling, which induced resistance to Notch inhibition, suggesting a dynamic crosstalk between the Hedgehog and Notch pathways. Combined, simultaneous targeting of Notch and Hedgehog pathways overcame the resistance to single agent-mediated Notch inhibition in glioblastoma and medulloblastoma models [30]. Hedgehog signaling has been shown to be necessary for the maintenance of glioblastoma and melanoma-initiating cells. Likewise, Notch signaling was found to be necessary for the maintenance of melanoma-initiating cells [10]. In light of these findings, our data suggest that combined blockade of the Hedgehog and Notch pathways, or perhaps WNT5 signaling, may be the optimal strategy for preventing the self-renewal of melanoma initiating cells.

Furthermore, we identified overexpression of GLI2 and SMO as a signature of resistance to smoothened inhibition in melanoma, as has been reported recently in human medulloblastoma using both NVP-LDE-225 and Vismodegib, an oral SMO inhibitor that is FDA-approved in advanced basal cell carcinoma. Two studies have reported amplifications of chromosomal regions containing GLI2 in Vismodegib and NVP-LDE-225-resistant medulloblastoma cell lines [31,32,33]. Our findings in the WM278 cell line suggest that, in melanoma harboring amplification of GLI2, hedgehog target genes are constitutively transcribed and upstream inhibition of hedgehog signaling via SMO inhibitors is ineffective. Our results indicate, that in those melanoma cells harboring GLI2 amplification, direct targeting of GLI2 is the best antitumor strategy. Pre-clinical evaluation of GLI-specific inhibitors is currently underway [34,35,36].

As NVP-LDE-225 enhanced the cytotoxic effects of single agent B-RAF inhibition, SMO inhibition may be effective in combination with vemurafenib in patients whose tumors express moderate levels of GLI2 and SMO. Based on the responses to NVP-LDE-225 in our melanoma cell line panel, it appears that a subset of patients whose tumors harbor either V600E B-RAF mutations or N-RAS mutation may benefit most from inhibition of SMO.

In summary, our pre-clinical studies support SMO as a therapeutic target in melanoma and offer insights into the clinical potential of this new treatment strategy both as a single agent and in combination with other targeted agents in metastatic melanoma patients.
